# Post-exposure effects of the piscicide 3-trifluoromethyl-4-nitrophenol (TFM) on the stress response and liver metabolic capacity in rainbow trout (*Oncorhynchus mykiss*)

**DOI:** 10.1371/journal.pone.0200782

**Published:** 2018-07-23

**Authors:** Oana Birceanu, Michael Patrick Wilkie

**Affiliations:** Department of Biology, Wilfrid Laurier University, Waterloo, Ontario, Canada; Universidade de Vigo, SPAIN

## Abstract

The piscicide 3-trifluoromethyl-4-nitrophenol (TFM) has been used to control invasive sea lamprey (*Petromyzon marinus*) populations in the Great Lakes for almost 60 years. Applied to rivers and streams containing larval lampreys, TFM seldom harms non-target fishes, but the effects of sub-lethal treatments on fish physiology are not well understood. We examined the effects of 9 h exposure to TFM on the stress axis and liver metabolic capacity of rainbow trout (*Oncorhynchus mykiss*) using *in vivo* and *in vitro* approaches. The fish that had been acutely exposed to TFM *in vivo* had increased plasma cortisol levels at 12 h post-treatment, but TFM exposure did not interfere with *in vitro* cortisol production in head kidney preparations. Subjecting trout to an acute handling stressor 12 h post-TFM exposure resulted in a relative attenuation of the plasma cortisol and glucose response compared to pre-stress levels. We conclude that routine TFM treatments can lead to elevations of plasma cortisol following exposure, plus a relative dampening of the stress response in rainbow trout, with high cortisol levels lasting at least 12 h post-treatment. Since the ability of the fish to produce cortisol and the liver metabolic capacity were not compromised following TFM exposure, it is likely that their ability to cope with other stressors is not altered in the long-term.

## Introduction

The piscicide (lampricide), 3-trifluoromethyl-4-nitrophenol (TFM), has been used for almost 60 years to control invasive sea lamprey (*Petromyzon marinus*) populations in the Great Lakes [1]. TFM applications are conducted in larval sea lamprey nursery streams and rivers, usually every 2–5 years, to reduce the numbers of parasitic juveniles that migrate to the Great Lakes, where they feed on economically important fishes [2]. To ensure maximum effectiveness, TFM is applied at 1.2–1.5 times the 9 h LC_99.9_, the minimum lethal concentration (MLC) needed to kill 99.9% of the larval sea lamprey [1]. The corresponding concentrations of TFM in the water seldom result in acute toxicity to non-target fishes, as the sea lamprey are much more sensitive to the chemical than non-target fishes and the pesticide rapidly degrades (2–5 days) in the environment [3]. However, there can occasionally be non-target effects in the field.

TFM exerts its toxicity in sea lampreys by uncoupling mitochondrial oxidative phosphorylation [4], which lowers ATP production and eventually leads to death [5–8]. The specificity of TFM to larval sea lamprey is based on their limited ability to detoxify the chemical. Detoxification occurs via the process of glucuronidation, a phase two biotransformation process that is used by most non-target fishes to detoxify TFM [9–12]. For this reason, most teleost fishes are much less sensitive to TFM [13].

Non-target fishes are routinely exposed to TFM during treatments [14], which can lead to significant TFM accumulation in their tissues, but the compound is rapidly cleared from the body following treatment due to their greater detoxification ability [15–17]. Because TFM uncouples oxidative phosphorylation, exposure to high concentrations over several hours results in significantly reduced high energy phosphagens and glycogen reserves in rainbow trout (*Oncorhynchus mykiss*) muscle and brain [12]. However, few other studies have examined the sub-lethal physiological effects of TFM on non-target fishes [18–20], and we are unaware of any that have addressed how TFM affects the stress response of non-target fishes. The goal of the present study was to establish if exposure to sub-lethal, environmentally relevant, concentrations of TFM affect the physiology of the rainbow trout, with a specific focus on the hypothalamic-pituitary-interrenal (HPI) axis, which mediates the stress response of fishes, and on liver metabolic capacity, since the liver is involved in detoxification of pesticides.

The activation of the HPI axis leads to the production of cortisol, and it has been shown to be targeted by various xenobiotics [21–26]. When a stressor is perceived, the hypothalamus signals the pituitary to release adrenocorticotropic hormone (ACTH) [27], which binds to the melanocortin-2 receptor (MC2R) on the inter-renal cells in the head kidney, activating the signaling cascade for cortisol production. Cholesterol, the raw material needed for steroid synthesis, is transported into the mitochondria by the steroidogenic acute regulatory protein (StAR), and then converted to pregnenolone via cytochrome P450 side-chain cleavage (P450scc) enzyme, the rate limiting step in cortisol biosynthesis [28]. This is followed by a series of reactions catalyzed by cytochrome P450 enzymes and hydroxysteroid dehydrogenases, leading to the production of cortisol. Because TFM interferes with mitochondrial oxidative ATP production in fishes [4], we predicted that routine lampricide treatments would impact the steroidogenic pathway in trout. To determine the effect of TFM on the ability of the fish to respond to subsequent stressors, we exposed trout to environmentally-relevant concentrations of TFM for 9 h, which were then subjected to an acute handling stressor 12 h following the TFM treatment, to ascertain if the stress response pathway and liver metabolic capacity were adversely affected.

## Material and methods

### Chemicals

Field formulation TFM (Clariant SFC GMBH WERK, Griesheim, Germany) was used for all experiments [35% active ingredient dissolved in isopropanol; provided courtesy of Fisheries and Oceans Canada (DFO), Sea Lamprey Control Centre, Sault Ste. Marie, ON, Canada], and TFM exposure concentrations were verified using precision TFM standards provided by DFO. The Glucose Liquicolor® assay kit (Stanbio Laboratory, Boerne, TX, USA) was used to measure tissue and plasma glucose levels. High-binding 96 well plates (Immulon HB, VWR Canlab, Mississauga) were used for an *in-house* cortisol enzyme-linked immunosorbent assay (ELISA), used in conjunction with a cortisol monoclonal antibody conjugated to horseradish peroxidase (East Coast Bio, ME, USA). The RiboZol^TM^ used for RNA extraction was obtained from AMRESCO (Solon, OH, USA), while the DNaseI was purchased from Sigma-Aldrich and the first strand cDNA synthesis kit was from Invitrogen (Burlington, ON, CA). The PerfeCTa® SYBR® Green FastMix® green fluorescent dye used for real-time quantitative PCR (RT qPCR) was purchased from Quanta Biosciences (Gaithersburg, MD, USA), while the 96-well plates were obtained from Bio-Rad (Mississauga, ON, CA). All other chemicals were purchased from Sigma-Aldrich (MO, USA).

### Experimental animals and holding

The experiments followed Canadian Council of Animal Care guidelines and were approved by the WLU Animal Care Committee. Rainbow trout juveniles (W = 64.3 ± 1.3 g, L = 18.1 ± 0.1 cm) were purchased from the Alma Research Station (ARS), Alma, ON, Canada. Fish were kept in a 750 L Living Stream (Frigid Units Inc., Toledo, OH) receiving Wilfrid Laurier University (WLU) well water (pH~ 8.0; titratable alkalinity ~ 200 mg CaCO3 L-1; hardness ~ 450 mg CaCO3 L-1; temperature 10–13 oC) at a rate of 2 L min^-1^. Fish were held under a 12 h light:12 h dark photoperiod and were fed 2% body weight three times per week. Fish were acclimated to these conditions for two weeks prior to the experiments. All experiments were conducted in the same WLU well water.

### Experimental protocol

#### Effects of *in vivo* TFM exposure on the HPI axis and liver metabolic capacity

**Fig 1:**
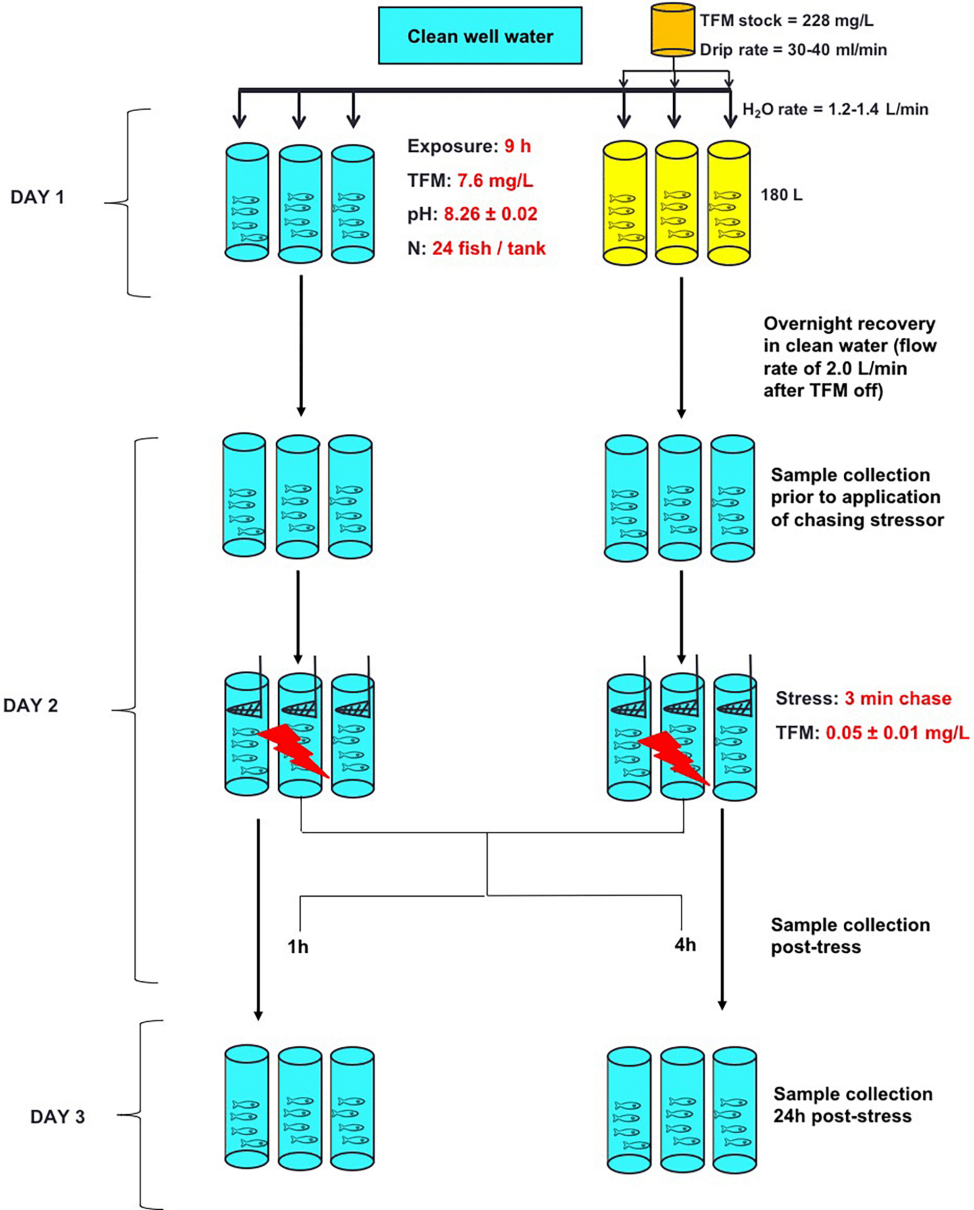
Experimental protocol—Effects of in vivo TFM exposure on the HPI axis and liver metabolic capacity. After an overnight acclimation to their tanks, fish (n = 3 tanks, n = 24 per tank) were exposed to the 9 h MLC of larval sea lamprey (nominal 7.6 mg L^-1^ TFM; Day 1) in continuously flowing Wilfrid Laurier University well water. Simultaneous controls (3 tanks, n = 24 fish per tank) were held under identical conditions, but not subjected to TFM exposure. At 9 h, the TFM drip was stopped and fish were allowed to recover overnight in tanks receiving continuously flowing well water (2.0 L min^-1^). On Day 2, tissues were collected from sub-samples of fish from each of the three control tanks and the three exposure tanks (n = 6 from each tank, time 0 h or pre-stress group). The remaining fish in all tanks were subjected to the application of a stressor (chasing and netting for 3 min [26]). The fish subjected to the stressor (n = 6 per time point per tank) were then sampled at 1 h and 4 h post-stress, with the remaining fish (n = 6) sampled at 24 h post-stress.

Food was withheld for 48 h prior to the beginning of the experiments. Twenty-four hours before the experiment, trout (n = 24 fish per tank) were placed in six 180 L, circular tanks continuously receiving WLU well water at a rate of 1.2–1.4 L min^-1^ and were left to acclimate overnight. The next day, fish in three tanks were exposed to 7.6 mg L^-1^ TFM (nominal) for 9 h, and the fish in the remaining three tanks served as un-exposed controls. The TFM exposure concentration chosen for the TFM exposure tanks was based on the previously determined 9 h LC_99.9_ of the larval sea lamprey (minimum lethal concentration, MLC) in Wilfrid Laurier University well water [6]. The exposure period to the MLC was 9 h to mimic the length that non-target fishes could be exposed to the MLC of sea lamprey during a typical TFM treatment, not including the time required for TFM to ramp-up to the target exposure concentration (Fig 1). At the beginning of the experiment, TFM was carefully added directly to each of the three exposure tanks from a concentrated stock (350 g L^-1^) to achieve the nominal concentration of 7.6 mg L^-1^ in the tank. Thereafter, the target concentration was maintained by a drip set-up using a pump (FMI Q-pump, Model QG150; Fluid Metering Inc., NY, USA) supplied with a TFM stock solution (228 mg TFM L^-1^), at a drip rate of 30–40 ml min^-1^, which mixed with the incoming well water (flow rate = 1.2–1.4 L min^-1^). All TFM solutions were kept in the dark to prevent light degradation during the exposure [3]. During the 9 h TFM exposure period, water samples (7 ml) were collected hourly followed by immediate spectrophotometric quantification of TFM (within 5 minutes) at a wavelength of 395 nm, as previously described [6,12].

After the 9 h exposure, the drip was stopped, and water flow was increased to 2.0 L min^-1^ to flush TFM from the tanks. TFM levels in the exposure tanks were monitored for an additional 5 h, to track the elimination of TFM from the experimental systems (S1 Fig). The fish were then left to recover overnight. After 12 h from when the TFM drip was stopped, sub-sets of trout (n = 6 per tank, total of n = 18 fish per time point) from each of the three control and three TFM tanks were quickly removed (pre-stress treatment) and euthanized with a lethal dose of tricaine methanesulfonate (MS222, Syndel, Port Alberni, BC, CA; 1.5 g L^-1^) buffered with 3.0 g L^-1^ NaHCO_3_. Immediately after the removal of the pre-stress group, the remaining fish (TFM-exposed and controls) were subjected to a chasing stressor, as previously described [26]. Briefly, fish were chased with a net and were repeatedly scooped (40 times) out of the water over 3 minutes, after which they were allowed to recover. All fish underwent the stress protocol before 9:30 a.m. Following the application of the stressor, the fish (n = 6 fish per time point, total of n = 18 fish per time point per treatment) were quickly removed from the tank, and were terminally sampled at 1, 4, and 24 h post-stress, and blood and tissues were collected at each time point for analysis. All fish were treated as individuals for the analysis, for a total of n = 18 fish per time point, per treatment.

#### Effects of TFM on *in vitro* cortisol production in the head kidney

A separate group of rainbow trout juveniles (n = 16, W = 92.7 ± 4.0 g, L = 20.4 ± 0.4 cm) were either exposed to a nominal TFM concentration of 7.6 mg L^-1^ (n = 8 fish per tank) for 9 h or kept in clean water (controls; n = 8 fish per tank), as previously described. After the TFM drip was stopped, fish were left to recover overnight, as described above. The next day, fish were placed in a lethal dose of MS222, bled by caudal severance and their head-kidneys were quickly extracted and placed in ice-cold Hank’s medium (composition in mmol L^-1^: NaCl = 137, KCl = 5.4, Na_2_HPO_4_ = 0.25, KH_2_PO_4_ = 0.44, MgSO_4_·7H_2_O = 0.8 and 5.0 of each HEPES, HEPES-Na, NaHCO_3_ and glucose; pH 7.63). The tissues were minced with a razor blade, washed in Hank’s medium 3-times, and each liver was equally divided into 3 wells on a 24 well plate and fresh 2 mL Hank’s medium was added. The plate was incubated at 13°C for 2 h, with gentle rocking, to allow the tissue to recover from the mincing and washing protocol. After the recovery period, the used Hank’s medium was removed, followed by the addition of fresh medium to well #1 (for determination of basal tissue cortisol levels) or medium containing adrenocorticotropic hormone (ACTH; 0.5 IU ml^-1^ in well #2) or 8-bromo-cyclic AMP (8BR; 0.5 mmol L^-1^ in well #3). The tissues were then incubated at 13°C for 4 h with gentle rocking. At the end of the experiment, the medium was placed in centrifuge tubes and was immediately frozen on dry ice. The tissues from each condition (basal, ACTH and 8BR) were placed in pre-weighed centrifuge tubes and frozen on dry ice. The weight of the tissues was determined by subtracting the tube weight from the total tube + tissue weight. All tissues and media were kept at -80°C until analysis.

### Analytical techniques

#### Tissue and blood collection

After each fish was anaesthetized, the caudal peduncle was severed using a razor blade (caudal severance), and the drops of blood collected into a 1.5 ml microcentrifuge tube coated with 5 mmol L^-1^ EDTA in 1× phosphate buffer saline (PBS; in mmol L^-1^: NaCl = 137.0, KCl = 2.7, KH_2_PO_4_ = 1.8, Na_2_HPO4·12 H_2_O = 10.0; pH 7.4). The tubes were then centrifuged at 5000 × *g* at room temperature and the plasma removed and placed in a separate microcentrifuge tube that was immediately frozen on dry ice. The liver was removed and placed in appropriately labelled tin foil, while the head-kidney was removed with a spatula and placed in 1.5 mL microcentrifuge tubes. Immediately after collection, all tissues were frozen on dry ice and placed in a -80°C freezer until analysis.

#### ELISA development for measuring plasma and media cortisol levels

Cortisol levels in the plasma and the media were measured using an *in-house* competitive enzyme-linked immunosorbent assay (ELISA), based on the protocol of Yeh et al. (2013) [29], with the modifications of Faught et al. [30].

#### Glucose, protein and lactate determination

Tissue and plasma glucose levels were measured colorimetrically using the Glucose Liquicolor assay kit (Kit No. 1070–125, Stanbio Laboratory, Boerne, TX, USA), by following the manufacturer’s instructions. Liver protein was determined using the bicinchoninic acid (BCA) method [31] on the 96-well plate spectrophotometer, with bovine serum albumin as the standard, using a commercial kit (G-Biosciences, Geno Technology, Inc., St. Louis, MO, USA). Plasma lactate levels were determined enzymatically (lactate dehydrogenase), as previously described [12].

#### Liver glycogen and metabolic capacity

Glycogen levels in the liver were determined by measuring glucose levels before and after amyloglucosidase digestion, as previously described [32,33]. Liver glycogen and glucose were normalized to tissue weight and expressed as μmol mg^-1^ wet weight.

The metabolic capacity of the liver was analyzed by monitoring enzyme activity via continuous (10 min) UV spectroscopy at 340 nm, at 22°C, using a plate spectrophotometer, as previously described [24,34,35]. Enzyme activity was normalized to protein content and it was expressed as μmol min^-1^ mg^-1^ protein. All enzymatic reactions were conducted in 50 mmol l^-1^ immidazole buffer (pH 7.4) and the conditions were as follows (final concentrations in the wells are shown):

- Hexokinase (HK)–in mmol l^-1^: 1.0 glucose, 5.0 MgCl_2_, 10.0 KCl, 0.25 NADH, 2.5 phosphoenol pyruvate; 20.0 U ml^-1^ lactate dehydrogenase, 2.5 U ml^-1^ pyruvate kinase; the reaction was started with 1 mmol l^-1^ ATP.
- Glucokinase (GK)–in mmol l^-1^: 20.0 glucose, 5.0 MgCl_2_, 10.0 KCl, 0.25 NADH, 2.5 phosphoenol pyruvate; 20.0 U ml^-1^ lactate dehydrogenase, 2.5 U ml^-1^ pyruvate kinase; the reaction was started with 1 mmol l^-1^ ATP.
- Pyruvate kinase (PK)–in mol l^-1^: 10.0 MgCl_2_, 30.0 KCl, 0.12 NADH, 2.5 ADP; 10.0 U ml^-1^ lactate dehydrogenase; the reaction was started with 2.5 mmol l^-1^ phosphoenol pyruvate.
- Lactate dehydrogenase (LDH)– 0.12 mmol l^-1^ NADH; the reaction was started with 1.0 mmol l^-1^ pyruvic acid.
- Phosphoenolpyruvate carboxykinase (PEPCK)– 1.0 MnCl_2_, 20.0 NaHCO_3_, 0.12 NADH, 0.5 phosphoenol pyruvate; the reaction was started with 0.2 mmol l^-1^ deoxyguanosine diphosphate.
- Alanine aminotransferase (AlaAT)–in mmol l^-1^: 0.12 NADH, 200 L-alanine, 0.025 pyridoxal 5-phosphate; 12.0 U ml^-1^ lactate dehydrogenase; the reaction was started with 10.5 mmol l^-1^α-ketoglutarate.
- Aspartate aminotransferase (AspAT)—in mmol l^-1^: 0.12 NADH, 7.0 α-ketoglutarate, 0.025 pyridoxal 5-phosphate; 8.0 U ml^-1^ malate dehydrogenase; the reaction was started with 40.0 mmol l^-1^ aspartate.

#### RNA extraction and first strand cDNA synthesis

Total RNA from the head-kidney was extracted using RiboZol™ Reagent, by following the manufacturer’s protocols. RNA extraction, along with DNase treatment and first strand cDNA synthesis were carried out as previously described [33]. RNA quality was assessed by determining the 260/280 ratio of the re-suspended RNA pellet, using a Nanodrop8000 Spectrophotometer (Thermo Scientific, Wilmington, DE, USA). A negative control (RNase-free water only) was run to test for contamination of the solutions used for RNA extraction (RNA negative control) and those used for cDNA synthesis (cDNA negative control).

#### Primers and quantitative real-time polymerase reaction

Primers were designed using MC2R, StAR and P450scc sequences previously published for rainbow trout (Table 1) [26] and were purchased from Integrated DNA Technolgies (Iowa, USA). The mRNA abundance was analyzed using the PerfeCTa SYBR Green FastMix green fluorescent dye (Quanta Biosciences, Gaithersburg, MD, USA) with the Bio-Rad C1000 Touch Thermal Cycler with CFX96™Real-Time System. The complete thermocyle was as follows: one cycle of 94°C for 2 min, 40 cycles [95°C for 30 sec, Annealing Temperature for 30 sec], 72°C for 10 min, followed by the melt curve. A melt curve (from 60–90°C in 0.5°C increments every 30 sec), along with the negative RNA and a negative cDNA control were used to ensure that a single product was amplified and that there was no contamination of the solutions. Copy number of each gene was determined by using relative standard curves, where the standards were made by serially diluting a pool (mix of 1 μl cDNA from each sample) of cDNA made from all the samples. All samples were assessed for the gene of interest, in duplicate, while the standard curve was run in triplicate. The relative transcript levels were determined by quantitative real-time PCR, as previously described [21] and each was normalized to the geometric mean of two reference genes, elongation factor 1 alpha and 18s ribosomal RNA (Table 1).

**Table 1:** Primer sequences.

Gene of interest	Sequence	Amplicon size (bp)	Accession #	Annealing temperature (˚C)
MC2R	F: 5′-GAGAACCTGTTGGTGGTGGT-3′	105	EU119870.1	60
	R: 5′-GAGGGAGGAGATGGTGTTGA-3′			
StAR	F: 5′-TGGGGAAGGTGTTTAAGCTG-3′	101	AB047032.2	60
	R: 5′-AGGGTTCCAGTCTCCCATCT-3′			
P450scc	F: 5′-GCTTCATCCAGTTGCAGTCA-3′	140	S57305.1	60
	R: 5′-CAGGTCTGGGGAACACATC-3′			
EF1α	F: 5′-CATTGACAAGAGAACCATTGA-3′	95	AF498320.1	56
	R: 5′-CCTTCAGCTTGTCCAGCAC-3′			
18sRNA	F: 5′-TGCGGCTTAATTTGACTCAACA-3′	117	AF243428.2	55
	R: 5′-CAACTAAGAACGGCCATGCA-3′			

Forward (F) and reverse (R) sequences, amplicon size, accession number and annealing temperature for the primers used in real-time quantitative PCR.

### Calculations and statistical analysis

Fish were treated as individuals for all the parameters measured. Because of the size of the fish used for the *in vivo* experiments and because some of the samples had to be re-measured for the various parameters that we report here, there was not enough tissue available to run all of the analysis with all 18 fish. Therefore, the actual numbers of individuals used for the analysis is represented in the brackets in the figure legend for each figure. For the gene mRNA abundance measured as part of the *in vivo* experiment, head kidneys from 2 fish from each replicate tank at each time point were analyzed, for a total of n = 6 individuals (2 from each replicate tank). There were no statistically significant differences between the tanks for all the parameters measured, as denoted by a one-way ANOVA. For the *in vitro* exposures, there were a total of 8 fish in the control tank and 8 fish in the TFM tank.

Statistical analysis was performed using IBM SPSS Statistics 23, while the figures were made using SigmaPlot 11.0 software (Systat Software Inc., San Jose, CA, USA). All data are shown as the mean ± standard error of the mean (S.E.M.). A nested model ANOVA, with the Bonferroni adjustment for multiple comparisons, was used for all *in vivo* experiments. The syntax modification employed in SPSS for the nested model ANOVA is listed in supplementary material (S1 Table). A mixed-model ANOVA used for the *in vitro* medium cortisol data. A probability level of p < 0.05 was considered significant. The data was log-transformed wherever necessary to meet the assumptions of the ANOVA and only non-transformed data are shown. For figures showing percent control, the S.E.M. was used as reference, as it is not a true reflection of the data variance. Raw data is available in Supplementary Material (S1 File).

## Results

### TFM levels in the water

Actual (measured) TFM concentrations in the exposure water are reported in Table 2, and there were no mortalities recorded during exposure, recovery or the post-stress period. Additional information on individual tank water quality parameters is provided in Table 2.

**Table 2:** Water parameters during TFM treatments for the *in vivo* and *in vitro* experiments.

Experiment	Treatment	Tank #	Measured TFM (mg/L)	pH	Temperature
*In vivo*	TFM	1	8.06 ± 0.13	8.30 ± 0.00	12.60 ± 0.06
		2	7.31 ± 0.28	8.24 ± 0.02	12.60 ± 0.10
		3	8.14 ± 0.12	8.26 ± 0.02	12.50 ± 0.00
	Control	1	0.53 ± 0.27	8.20	12.3
		2	0.53 ± 0.27	8.29	12.8
		3	0.03 ± 0.01	8.33	12.5
*In vitro*	TFM	1	7.84 ± 0.29	8.03 ± 0.01	13.0 ± 0.00
	Control	1	0.00 ± 0.00	8.05 ± 0.03	13.0 ± 0.00

The parameters reported here were measured in the experimental tanks prior to, during and at the end of the TFM exposure, and samples were collected from both the bottom and the top of the exposure tanks, in both sets of experiments. pH and temperature measurements were taken only once in the control tanks for the *in vivo* experiments, at the beginning of the experiment. TFM numbers in control tanks for the *in vivo* experiments represent background measurements. All data shown here is without background subtracted. Data presented as mean ± S.E.M.

### Plasma stress indices

**Fig 2:**
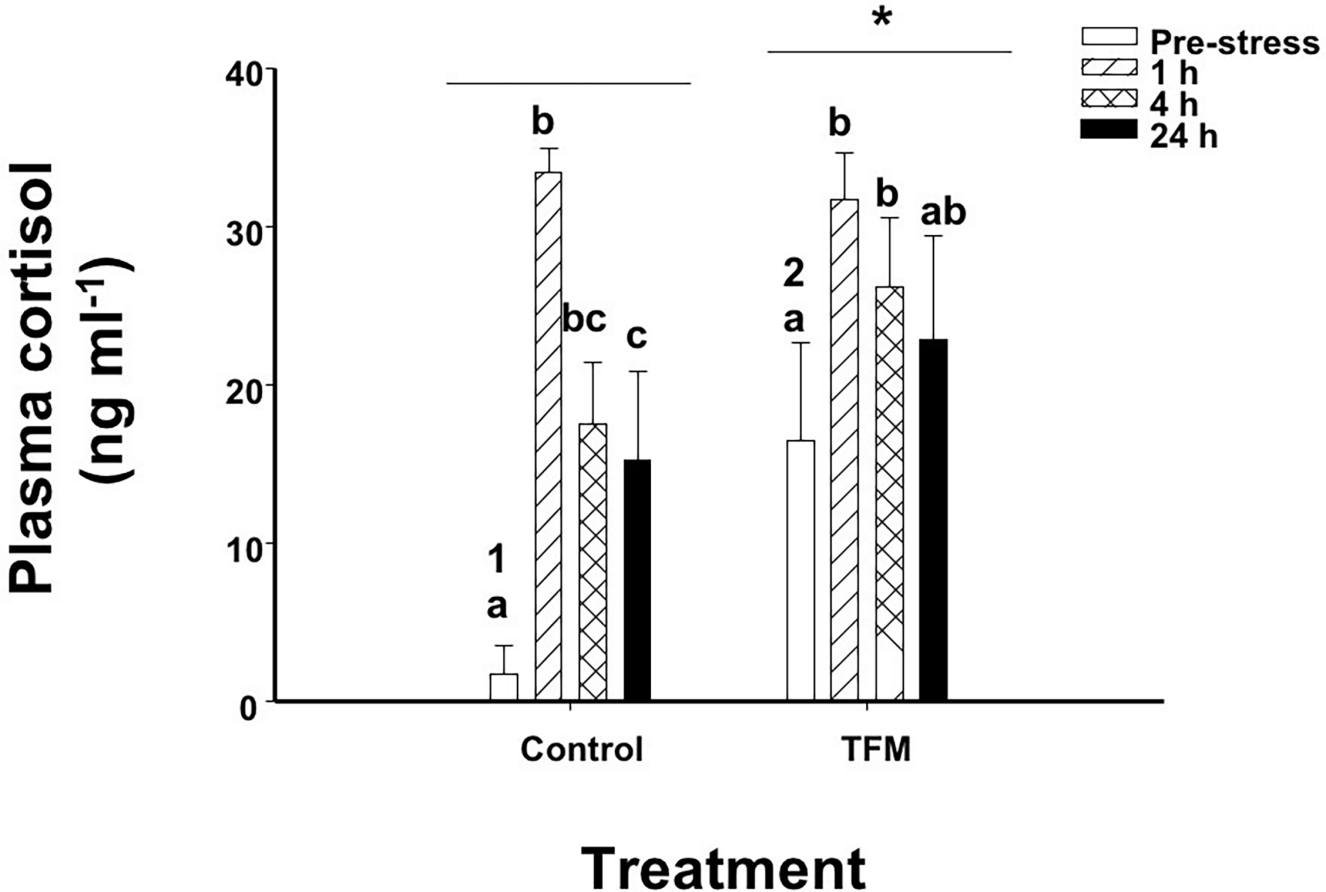
Plasma cortisol. Effects of prior exposure of rainbow trout to TFM on stress-induced increases in plasma cortisol. Significant differences are indicated by letters (between time points within a treatment), numbers (between treatments within a given time point) or * (overall between treatments). Data presented as mean ± S.E.M. (n = 7–11, *p* < 0.05; nested-model ANOVA with the Bonferroni correction).

The concentrations of cortisol in unstressed (pre-stress) control fish not exposed to TFM were approximately 1.77 ± 1.76 ng mL^-1^. In contrast, cortisol concentrations in TFM-exposed non-stressed fish were approximately 9-fold greater than controls the day after exposure to the chemical, averaging 16.5± 6.1 ng mL^-1^ (pre-stress; Fig 2). The response to handling stress (chasing and netting) in the control group was characterized by a significant ~10-fold increase in plasma cortisol at 1 h post-stress, and then dropped by 50% by 4 h, stabilizing at concentrations that were 4- to 5-fold greater than in the unstressed controls (Fig 2). The fish exposed to TFM 12 h earlier and then subjected to handling stress, experienced a significant 2-fold elevation in cortisol after 1 h compared to the non-stressed TFM-exposed fish. Cortisol levels remained elevated in the TFM exposed fish through the entire 24 h post-stress period (Fig 2). Overall, plasma cortisol levels were significantly higher in the TFM-exposed fish than in the non-exposed controls.

**Fig 3:**
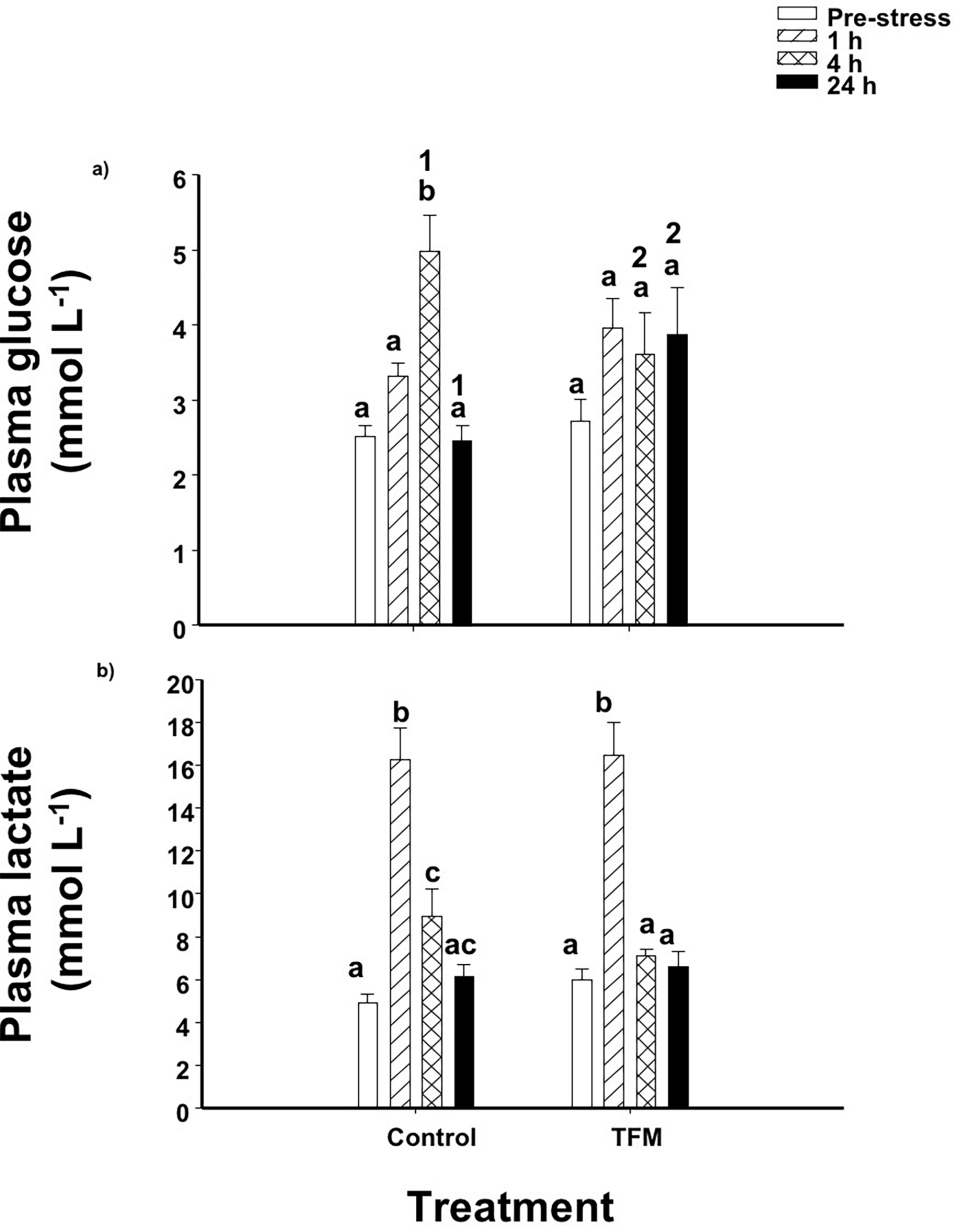
Plasma glucose and lactate. Effects of prior TFM exposure on plasma (a) glucose and (b) lactate levels in rainbow trout, following exposure to an acute handling stress. Significant differences are indicated by letters (between time points within a treatment) and numbers (between treatments within a given time point). Data presented as mean ± S.E.M. (n = 11–13, *p* < 0.05; nested-model ANOVA with the Bonferroni correction).

Plasma glucose levels in non-TFM exposed control fish significantly increased by 2-fold by 4 h post-stress when compared to the unstressed fish, before returning to pre-stressor concentrations by 24 h (Fig 3A). In the TFM-exposed fish, plasma glucose concentration remained unaltered following handling stress. However, at 4 h, glucose levels in controls were approximately 1.5-fold higher than in the TFM-exposed individuals, while at 24 h following the handling stress, glucose levels in control fish had decreased to non-stressed levels. In the TFM group, plasma glucose remained 1.5-fold higher than in the control fish (Fig 3A).

The plasma lactate concentrations in controls and TFM-exposed animals were not significantly different from one another prior to subjecting the animals to handling stress. In both groups, handling stress resulted in a marked 4-fold increases in plasma lactate at 1 h post-stress. While plasma lactate levels remained slightly (2-fold) elevated at 4 h in the non-exposed control fish, they had returned to pre-stressor levels by 4 h in the TFM-exposed fish (Fig 3B).

### Relative mRNA abundance of steroidogenic genes in the head kidney

**Fig 4:**
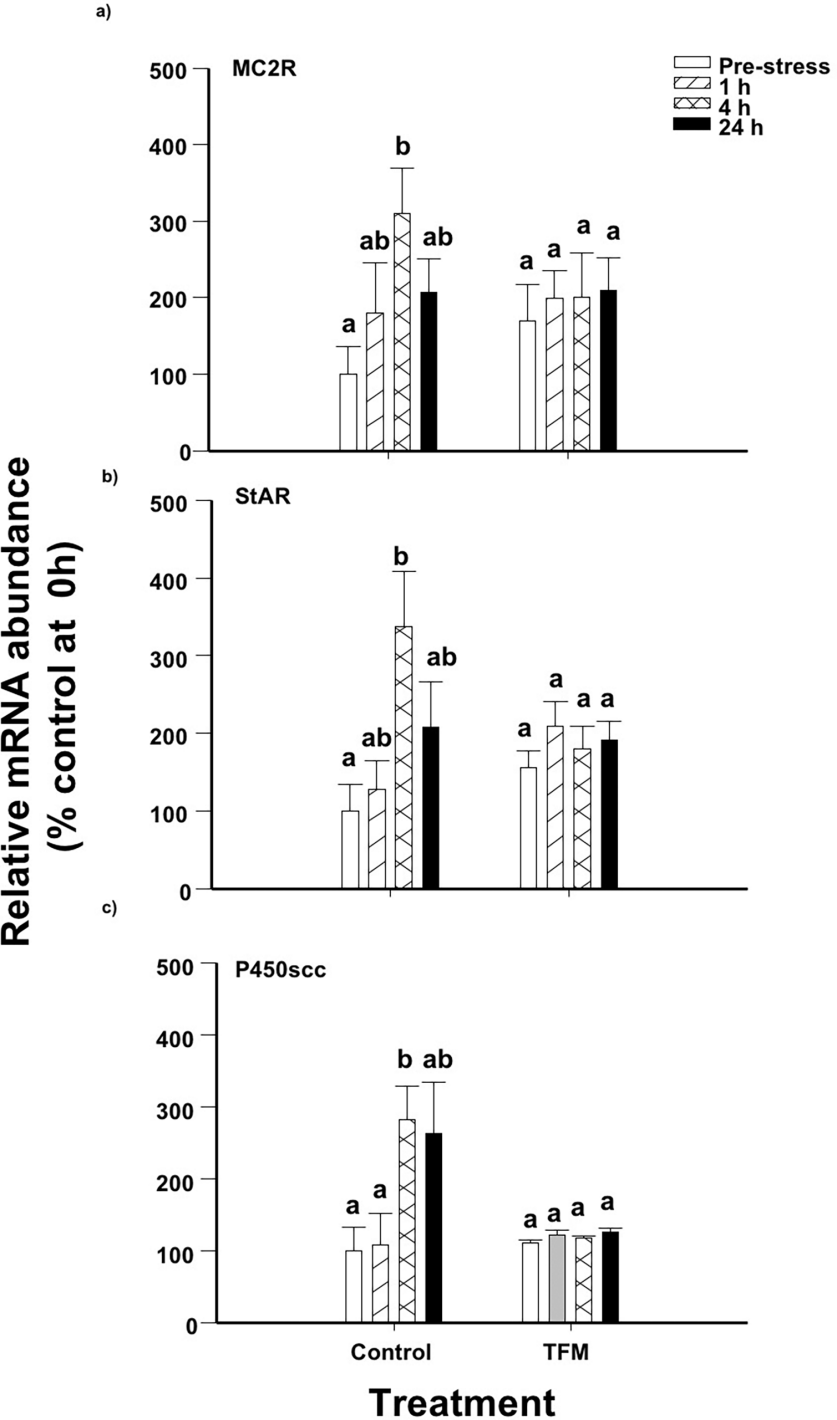
Head kidney steroidogenic gene mRNA abundance. Effects of prior exposure of rainbow trout to TFM on stress-induced increases on head kidney (a) MC2R, (b) StAR and (c) P450scc mRNA abundance. Data was normalized to total RNA and expressed as % control at 0 h. Data points sharing the same letter are not significantly different within one treatment. Data presented as mean ± S.E.M. (n = 5–6; *p*<0.05; nested-model ANOVA with the Bonferroni correction).

Following a handling stress, MC2R mRNA abundance in the head kidney in control fish not exposed to TFM, increased by approximately 3-fold after 4 h when compared to non-stressed fish, and returned to non-stressed levels at 24 h (Fig 4A). In contrast, there were no statistically significant changes in the MC2R mRNA abundance in the TFM-treated fish following handling stress and the profile was not significantly different than observed in controls. Head kidney StAR mRNA levels increased by 3-fold at 4 h post-stress in the control fish post-stress, and the levels returned to those of the controls after 24 h (Fig 4B). However, handling stress did not result in significant changes in StAR mRNA abundance in the TFM-treated fish and there was no overall effect of prior exposure to TFM on StAR mRNA abundance when compared to control fish. Similarly, P450scc mRNA abundance, which significantly increased by approximately 3-fold in non-TFM exposed control fish at 4 h compared to pre-stressor levels, did not significantly change in the TFM-exposed fish subjected to the handling stressor (Fig 4C). Overall, there were no significant effects of the prior TFM exposure on P450scc mRNA abundance when compared to controls.

### *In vitro* cortisol production in the head kidney

Actual TFM concentrations in the exposure water ae reported in Table 2, and there were no mortalities recorded during exposure, recovery or the post-stress period. Also refer to Table 2 for water quality parameters for each holding tank.

**Fig 5:**
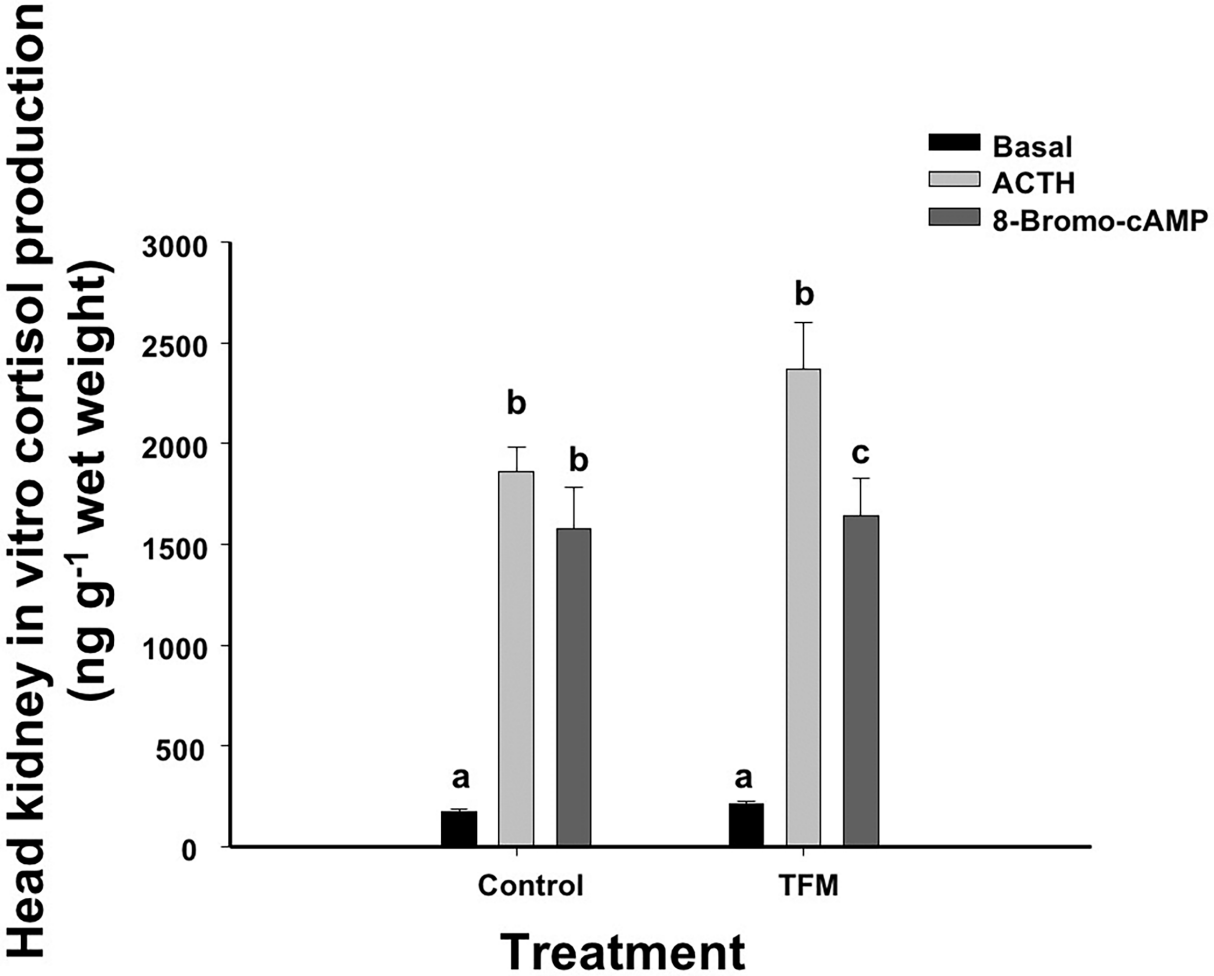
Head kidney *in vitro* cortisol production. Effects of prior TFM exposure on *in vitro* cortisol production in head kidney preparations following a 4 h challenge with either ACTH (0.5IU/ml) or 8-bromo-cyclic AMP (8-Bromo-cAMP; 0.5 mmol L^-1^). Data points sharing the same letter are not significantly different within one treatment. Data presented as mean ± S.E.M. (n = 8; *p* < 0.05; mixed-model ANOVA). There were no treatment effects or interactions noted on *in vitro* cortisol production.

There were no differences in *in vitro* cortisol production between the fish exposed to TFM or the non-exposed control fish. In both cases, respective media cortisol concentrations increased by 11-fold when the head kidney tissue was stimulated with ACTH (Fig 5). Following incubation of the head kidney preparation with 8BR, cortisol concentration increased by 8-9-fold in the control and TFM-exposed fish, again demonstrating that there was no TFM effect on cortisol production.

### Liver glycogen and metabolic capacity

**Fig 6:**
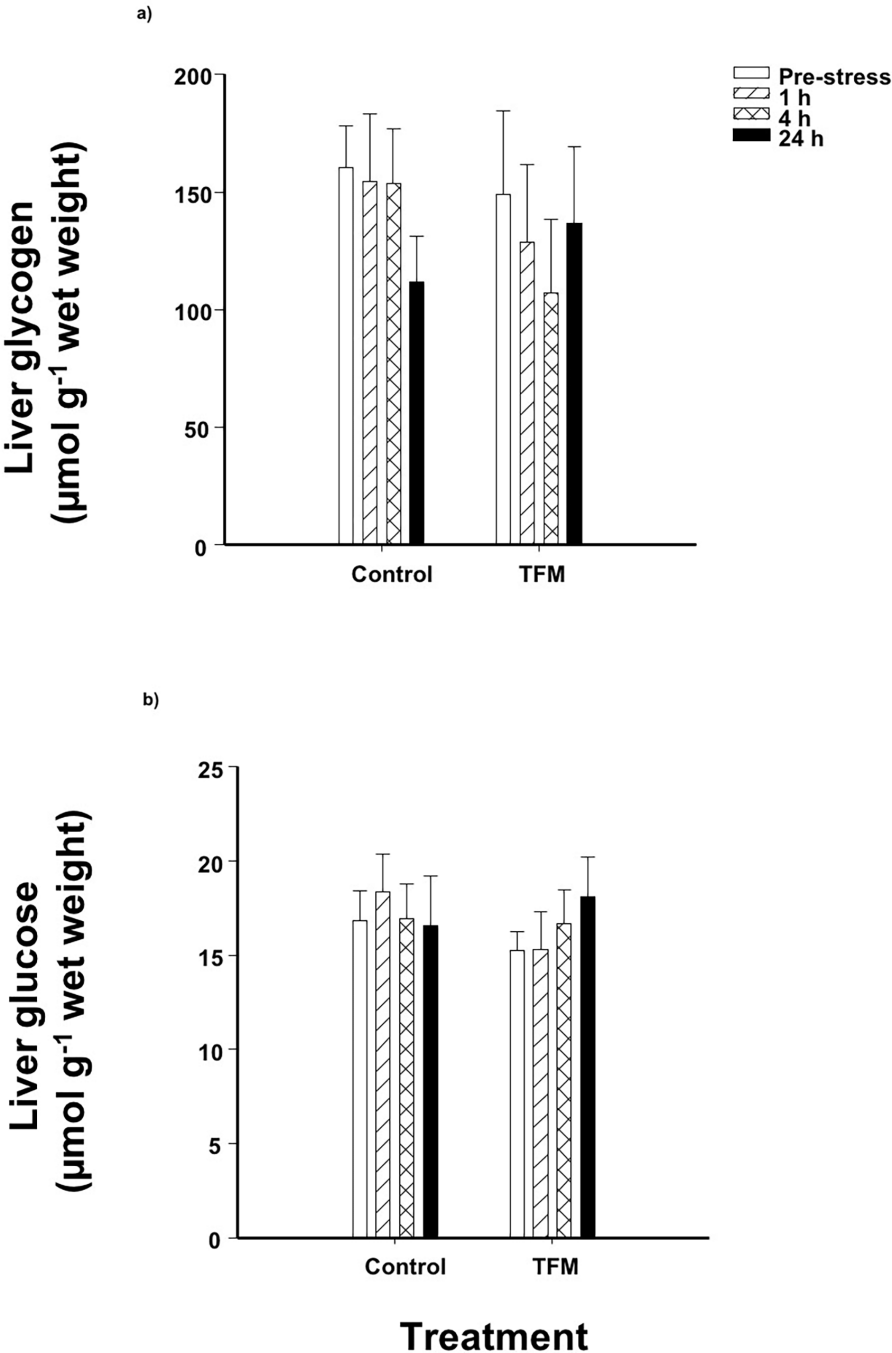
Liver glycogen and glucose. Effects of prior exposure to TFM on liver (a) glycogen and (b) glucose concentrations of rainbow trout, following exposure to an acute handling stress. There were no significant effects of treatment or time and there were no interactions. Data presented as mean ± S.E.M. (n = 11–13; *p*<0.05; nested-model ANOVA with the Bonferroni correction).

Glycogen stores in fish that had been exposed to TFM were not significantly different from the non-exposed controls, and the application of a subsequent handling stressor post-treatment did not affect liver glucose and glycogen stores in either the control or the TFM-exposed fish (Fig 6). The activities of LDH and AspAT were significantly lower at 24 h post-stress (36h post-exposure) compared to 4 h and unstressed fish, respectively, and this effect was seen in both controls and TFM-treated fish. There were no effects of prior TFM exposure on enzyme activity in the fish liver (Table 3).

**Table 3:** Liver metabolic capacity in rainbow.

	Treatment	Post-stress enzyme activity (μmol min^-1^ mg^-1^ protein)				Time effect (p<0.05)
		Pre-stress	1 h	4 h	24 h	
**LDH**	Control	143.38 ± 2.57	145.05 ± 3.11	149.78 ± 5.20	135.68 ± 7.33	24 h < 4 h
	TFM	143.53 ± 4.16	143.52 ± 4.60	144.85 ± 3.31	133.43 ± 2.35	24 h < 4 h
**HK**	Control	3.55 ± 0.10	3.66 ± 0.12	3.75 ± 0.12	3.39 ± 0.12	
	TFM	3.84 ± 0.18	3.88 ± 0.21	3.69 ± 0.13	3.43 ± 0.10	
**GK**	Control	3.29 ± 0.14	3.51 ± 1.17	3.94 ± 0.20	3.48 ± 0.13	
	TFM	3.55 ± 0.19	3.76 ± 0.21	3.65 ± 0.16	3.39 ± 0.07	
**PK**	Control	13.38 ± 1.32	13.48 ± 0.86	12.93 ± 1.09	12.95 ± 1.02	
	TFM	13.27 ± 1.12	13.99 ± 1.08	13.00 ± 1.19	13.65 ± 1.13	
**PEPCK**	Control	1.26 ± 0.05	1.35 ± 0.04	1.34 ± 0.04	1.30 ± 0.04	
	TFM	1.32 ± 0.06	1.37 ± 0.04	1.37 ± 0.05	1.33 ± 0.03	
**AlaAT**	Control	19.32 ± 1.85	19.56 ± 1.62	17.34 ± 1.12	20.19 ± 1.88	
	TFM	20.38 ± 1.27	21.80 ± 0.79	18.89 ± 1.49	19.51 ± 0.88	
**AspAT**	Control	16.06 ± 1.09	15.06 ± 0.87	14.11 ± 1.10	13.45 ± 0.88	24 h < 0 h
	TFM	16.26 ± 1.47	16.00 ± 1.18	14.91 ± 1.06	12.89 ±0.65	24 h < 0 h

Enzyme activities were measured in trout pre- (0 h) and post-exposure (1,4, 24 h) to an acute handling stressor, after recovery in clean water from either control (no TFM) or TFM treatment (nominal concentration of 7.6 mg L^-1^). Data presented as mean ± S.E.M. (n = 11–13; *p*<0.05; nested-model ANOVA with the Bonferroni correction). Lactate dehydrogenase (LDH), hexokinase (HK), glucokinase (GK), pyruvate kinase (PK), phosphoenylpyruvate carboxykinase (PEPCK), alanine aminotransferase (AlaAT) and aspartate aminotransferase (AspAT). N = 11–13 individual fish per measurement.

## Discussion

### Higher *in vivo* cortisol levels post-TFM treatment and the role of the liver

The present study shows that *in vivo* exposure of rainbow trout to TFM, mimicking a typical lampricide treatment, caused an increase in plasma cortisol levels that persisted following the TFM treatment, but had no effect on head kidney cortisol production *in vitro*. The TFM-induced increase in cortisol *in vivo* in the present context could be advantageous to the fish short-term immediately after TFM exposure, as it allows them to cope with and recover from the exposure. Short-term elevations of cortisol would be advantageous because cortisol promotes glycogenolysis [36–39], making more glucose available for metabolic processes [36,37], as the fish rely on glycolysis to meet the demands of the body during and following TFM exposure [5–7,12]. In addition, glycogenolysis provides glucose-6-phosphate, which is then used as a substrate for glucuronic acid production, a major player involved in pesticide detoxification via glucuronidation [40].

Although the fish were not sampled immediately after TFM exposure, we presume that TFM led to elevated plasma cortisol levels during the 9 h exposure, and that TFM remained elevated over the ensuing 12 h that followed the termination of the exposure (pre-stressor). Indeed, exposure to other pesticides and xenobiotics induces similar prolonged elevations in cortisol, which are normally seen within the first hours of exposure [24,25,39,41]. Exposure to TFM also causes marked reductions in brain glycogen reserves of fishes as the fish rely more on glycolysis to make up for shortfalls in oxidative ATP supply [6–8]. Thus, hepatic glycogenolysis would augment glucose supply to the nervous system, which requires glucose to function [42], should the lampricide exposure be high enough. The fact that we did not find any disturbances in liver glycogen or liver glucose in the present study suggests that perhaps the exposure concentration of the lampricide was not high enough to elicit marked disturbances in liver energy reserves. In previous studies conducted by our group [6,12], we exposed rainbow trout and sea lamprey to their respective TFM LC_50_ (11.0 mg L^-1^ for trout and 4.8 mg L^-1^ for lamprey) and, similarly, did not record any reductions in the liver glycogen of the trout. In the sea lamprey, however, which are more sensitive to TFM, liver glycogen decreased by more than 80% by the end of the 12 h TFM exposure. Therefore, it appears that trout are able to cope with TFM at levels comparable to those typically used in the field.

Another advantage of short-term elevated cortisol levels could be related to the fact TFM detoxification using glucuronidation relies on glucose, which is needed to generate glucose-6-phosphate (G6P), a substrate required to produce the glucuronic acid needed for the detoxification of TFM to proceed by glucuronidation in the liver [40]. Cortisol has been shown to induce liver glycogenolysis in fish [37], and, therefore, the elevation of cortisol levels post-TFM exposure may be required to facilitate the supply of G6P substrate for glucuronic acid production and TFM elimination in the hours immediately following exposure. Most non-target fishes, rainbow trout in particular, do have a high capacity to detoxify TFM [11,16] and would rely on glucuronidation to eliminate the lampricide from the body [12, 43]. Moreover, TFM and TFM-glucuronide have been detected in the muscle fillet of fish up to 12 h following a routine TFM application [16], suggesting that detoxification still occurs for several hours following exposure to the lampricide.

Like glycogen reserves, the metabolic capacity of the liver was also unaffected by TFM exposure, as demonstrated by the lack of changes in the activity of glycolytic enzymes such as LDH, HK, GK and PK. In addition, the activity of PEPCK, a key enzyme in gluconeogenesis, and the activities of AlaAT and AspAT, two enzymes involved in amino acid catabolism, were also unaffected by TFM. It appears that the trout liver glycolytic capacity and its ability to break down proteins remained unaltered, in addition to exhibiting no changes in liver glycogen and glucose. This suggests that routine lampricide applications, at the MLC of the larval sea lamprey, have minimal metabolic disturbances on the rainbow trout,

### Response to an acute handling stress following the TFM treatment

The increase in cortisol at 1 h post-stress in both the controls (no TFM) and the TFM-treated fish suggests that the latter group was still able to respond to an acute stressor, even though their non-stressed (0 h) cortisol levels were much higher than in controls. The control, non-TFM exposed, fish had much greater relative increases in cortisol at 1 h post-stress, when compared to the TFM-exposed fish (~10-fold increase in controls vs. only a 2-fold increase in the TFM group), suggesting that TFM does act as a stressor on its own. Typically, following an acute stressor exposure, there is also an elevation in the mRNA abundance of the steroidogenic genes MC2R, StAR and P450scc [28,44,45]. This elevation in transcript abundance is noted at or following peak cortisol in plasma, at 1–4 h post-stressor and its level of increase is highly dependent on the intensity of the acute stressor [46]. While this response was noted in the head kidney of control (non-TFM exposed) fish in the current study, there were no elevations in the mRNA abundance of the steroidogenic genes in the head kidney of the TFM-exposed fish when compared to controls. This lack of increase in mRNA levels in steroidogenic genes is similar to that reported in salmonids exposed to chronic stress. Similarly, Fierro-Castro et al. [47] reported that following a 5-day chronic crowding stress, the mRNA levels of the steroidogenic genes in the head kidney in the crowded individuals remained unaltered when compared to control, uncrowded fish. As well, Madaro et al. [48] reported that exposure of Atlantic salmon (*Salmo salar* L.) parr to unpredictable chronic stress for 3 weeks did not lead to an elevation of transcript levels of MC2R or StAR 1 h after the application of a novel stressor. The authors suggested that perhaps the stressor frequency, along with sampling in the first days of the experiment, had caused an exhaustion of the HPI axis, leading to a decreased ability to respond to an additional stressor. It is, therefore, possible that previous exposure to the lampricide already led to the production of high levels of steroidogenic proteins in the kidney cells during and immediately following the exposure, since high cortisol levels were recorded just prior to the application of the chasing-netting stressor at 12 h post-TFM exposure (Fig 1). This would decrease the need to upregulate gene expression following the induction of a novel stressor, which is what the current study is suggesting. However, further studies are needed to confirm this hypothesis.

Liver glycogen and glucose levels remained unaffected by either the TFM treatment or the handling stressor. This is not surprising, because the trout liver is a large glycogen pool compared to other tissues, and it is mainly used to maintain glucose homeostasis during periods of prolonged starvation or chronic stress [49–52]. Vijayan and Moon [53] reported that an acute stressor exposure impacted liver glycogen only in trout that were starved for 30 days, but not in fed individuals, therefore highlighting the robustness of this energy storing organ. Because the trout in the present study were regularly fed, and only fasted 2 days prior to TFM-exposure, it likely explains the absence of any substantive change in liver glycogen following TFM exposure and a 3-min acute handling stress. It is therefore possible that liver glycogen stores could be more sensitive to TFM in fish that have been fasted for long periods in the field, if food resources are limited by factors such as season, or if TFM treatment coincides with other exogenous stressors that may affect fish metabolism (e.g. high temperature, high water flow, altered dissolved oxygen). Indeed, previous studies have shown that liver glycogen reserves are reduced following chronic exposure to metals or municipal waste effluent [24,25].

Although non-exposed control fish at 24 h post-acute stressor exposure had significantly lower cortisol levels than at 1 h post-stress, they remained higher than the non-stressed fish (pre-stressor). This prolonged elevation of cortisol has been previously reported in trout [35], and it is often attributed to repeated sampling from the same tank and reduced density, as low density in trout has been linked with increased aggression and, therefore, elevated cortisol [54]. In contrast to the control fish, however, it was intriguing to see that the circulating cortisol levels in the TFM-treated fish at 24 h post-stress remained elevated and were not significantly different than at their peak, 1 h post-stress levels. Chronically elevated cortisol in fish negatively impacts the endocrine response to stressors long-term in the progeny [55], and it also affects gamete quality and survivability [56], parental care [57] and social status [58,59]. It is possible that TFM exposure interferes with cortisol clearance from the blood, by either affecting the signaling pathway, or by impacting cortisol breakdown. Once cortisol is in the cell, it either binds to its receptor and it is activated, or it is metabolized through the action of reductases, oxidoreductases, cytochrome P-450 dependent hydroxylases [60] and via the processes of glucuronidation (5%) and sulfonation (95%) [61]. It is possible that the remaining TFM in the body was competing with cortisol for UDP-glucuronyltransferase, which facilitates glucuronidiation in the liver [46]. This would have potentially slowed cortisol catabolism in the liver 36 h post-treatment, but it seems unlikely because TFM is normally cleared from non-target fish muscle by 12 h post-exposure [16]. Future studies should examine the stress physiology of the fish during and immediately following TFM exposure, with a focus on the liver and detoxification pathways, to shed more light on cortisol dynamics and their long-term implications.

### *In vitro* cortisol production following TFM exposure

Through the *in vitro* experiment conducted in the present study, we have shown that the steroidogenic capacity in trout is not compromised by previous TFM exposure in the laboratory. Exposure of kidney preparations to either ACTH or 8-bromo-cyclic AMP led to comparable increases in cortisol production in fish, regardless of treatment. ACTH binds to the melanocortin-2 receptor (MC2R) on the inter-renal cells in the head kidney, leading to the activation of the signaling cascade for cortisol production, while 8-bromo-cyclic AMP is a cyclic AMP analog which activates protein kinase A, leading to the phosphorylation of StAR and to cholesterol translocation in the mitochondria for cortisol production. The current findings do suggest that these specific pathways, prior to the translocation of cholesterol in the mitochondria, remain unaffected by TFM exposure in trout.

### Implications of the current findings

The current study demonstrates that the effects of TFM on the stress response and HPI axis components of non-target rainbow trout are minimal following TFM exposure. Although TFM acted as a stressor on its own, leading to elevated plasma cortisol levels 12 h post-exposure, this elevation could have been advantageous short-term, as cortisol induced glycogenolysis, providing glucose for metabolic processes and substrates for detoxification. How long after TFM treatment these elevated cortisol levels persist in trout, and if other non-target fishes respond the same way to TFM, is currently unknown. Gaps remain in our knowledge of how TFM affects the stress responses of non-target fishes in the field, particularly during earlier, more sensitive, developmental stages [14]. As previously stated, chronically elevated cortisol in fish can negatively impact the endocrine response to stressors long-term, leading to effects on gamete quality, survivability, progeny performance and may impact the overall social status and parental care ability of the adults. In addition, TFM leads to an increased reliance on anaerobic glycolysis to make up for shortfalls in ATP supply [6,7,12], and, if applied in the presence of additional exogenous stressors (e.g. adverse environmental conditions or food deprivation), it is possible that a fishes’ability to handle such stressors could be compromised. Therefore, further research is required to develop a better understanding of how the HPI axis is influenced by exposure to routine TFM treatments, in the field, to recognize any possible adverse risks to non-target fishes and adopt measures to reduce or eliminate them.

## Supporting information

S1 Fig Depuration of TFM from the exposure tanks. TFM depuration rates in 2 out of the 3 tanks of fish exposed to nominal TFM of 7.6 mg L^-1^ *in vitro*. Note that TFM depuration rates were measured from the TFM concentration measured in the tank immediately after the drip was stopped. For an average TFM exposure level in the fish in each tank, refer to Table 2.
(PDF)

S1 Table Syntax modifications in IBM SPSS Statistics 23 used for the nested model ANOVA. Note that Y was the dependent variable (e.g. plasma cortisol, plasma glucose, mRNA abundance, etc.).
(PDF)

S1 File This file represents the raw data reported in this manuscript. (XLSX)
