# Supplementary material for: Post-exposure effects of the piscicide 3-trifluoromethyl-4-nitrophenol (TFM) on the stress response and liver metabolic capacity in rainbow trout (Oncorhynchus mykiss)
Source: PLoS One. 2018 Jul 23;13(7):e0200782. doi: 10.1371/journal.pone.0200782 (PMC6056040; doi:10.1371/journal.pone.0200782)
Supplement: S1 Fig — TFM depuration rates in 2 out of the 3 tanks of fish exposed to nominal TFM of 7.6 mg L-1 in vitro. Note that TFM depuration rates were measured from the TFM concentration measured in the tank immediately after the drip was stopped. For an average TFM exposure level in the fish in each tank, refer to Table 2. (PDF) [file pone.0200782.s001.pdf]

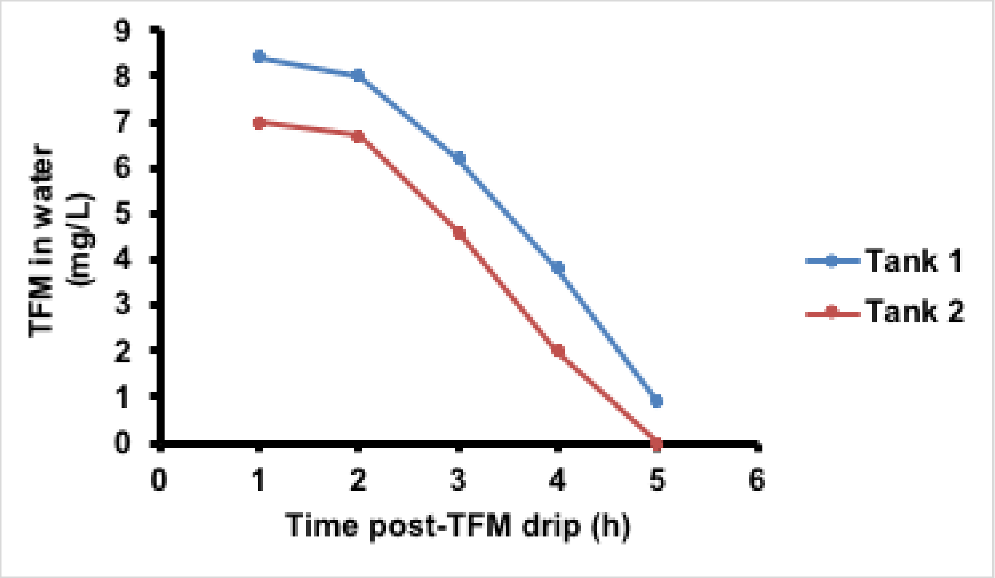

S1 Figure. Depuration of TFM from the exposure tanks. TFM depuration rates in 2 out of the 3 tanks of fish exposed to nominal TFM of 7.6 mg L^{-1} *in vitro*. Note that TFM depuration rates were measured from the TFM concentration measured in the tank immediately after the drip was stopped. For an average TFM exposure level in the fish in each tank, refer to Table 2.
