# Supplementary material for: Post-exposure effects of the piscicide 3-trifluoromethyl-4-nitrophenol (TFM) on the stress response and liver metabolic capacity in rainbow trout (Oncorhynchus mykiss)
Source: PLoS One. 2018 Jul 23;13(7):e0200782. doi: 10.1371/journal.pone.0200782 (PMC6056040; doi:10.1371/journal.pone.0200782)
Supplement: S1 Table — Note that Y was the dependent variable (e.g. plasma cortisol, plasma glucose, mRNA abundance, etc.). (PDF) [file pone.0200782.s002.pdf]

S1 Table. Syntax modifications in IBM® SPSS® Statistics 23 used for the nested model ANOVA. Note that Y was the dependent variable (e.g. plasma cortisol, plasma glucose, mRNA abundance, etc.).

```
VARIABLE LABELS Y 'Y'.
VARIABLE LABELS Time 'Time'.
VARIABLE LABELS Treatment 'Treatment'.
UNIANOVA
Y BY Treatment Time
/METHOD = SSTYPE(2)
/INTERCEPT = INCLUDE
/POSTHOC = Treatment Time ( BONFERRONI )
/EMMEANS = TABLES(Treatment) COMPARE ADJ(BONFERRONI)
/EMMEANS = TABLES(Time) COMPARE ADJ(BONFERRONI)
/PRINT = DESCRIPTIVE ETASQ
/CRITERIA = ALPHA(.05)
/DESIGN = Treatment Time(Treatment).
```
